# An algorithm of image mosaic based on binary tree and eliminating distortion error

**DOI:** 10.1371/journal.pone.0210354

**Published:** 2019-01-07

**Authors:** Zhong Qu, Xue-Ming Wei, Si-Qi Chen

**Affiliations:** 1 School of Software Engineering, Chongqing University of Posts and Telecommunications, Chongqing, People’s Republic of China; 2 College of Computer Science and Technology, Chongqing University of Posts and Telecommunications, Chongqing, People’s Republic of China; 3 Chongqing Engineering Research Center of Software Quality Assurance, Testing and Assessment, Chongqing, People’s Republic of China; Huazhong University of Science and Technology, CHINA

## Abstract

The traditional image mosaic result based on SIFT feature points extraction, to some extent, has distortion errors: the larger the input image set, the greater the spliced panoramic distortion. To achieve the goal of creating a high-quality panorama, a new and improved algorithm based on the A-KAZE feature is proposed in this paper. This includes changing the way reference image are selected and putting forward a method for selecting a reference image based on the binary tree model, which takes the input image set as the leaf node set of a binary tree and uses the bottom-up approach to construct a complete binary tree. The root node image of the binary tree is the ultimate panorama obtained by stitching. Compared with the traditional way, the novel method improves the accuracy of feature points detection and enhances the stitching quality of the panorama. Additionally, the improved method proposes an automatic image straightening model to rectify the panorama, which further improves the panoramic distortion. The experimental results show that the proposed method cannot only enhance the efficiency of image stitching processing, but also reduce the panoramic distortion errors and obtain a better quality panoramic result.

## 1. Introduction

Image mosaic is the integration of multiple images with overlapping regions into a non-distorted, high-resolution panoramic image [1–2]. Improving the real-time and splicing quality of image mosaic has become an important research agenda in the field of computer vision and graphics [3–4].

In the field of image stitching, related algorithms can be divided into two kinds: image mosaic based on gray level information, and image mosaic based on the features [5]. The former, based on the gray level information, is to calculate the similarity degree of the two images by using the pixel value of the image to be spliced. This is done to determine the overlapping area of the splicing and realize the splicing of the image. However, this method is computationally intensive and not robust enough [6]. The latter, based on the features, is to extract the relevant feature information in the image, then match the features of the two images to obtain the mapping relation between the images. Most of the algorithms are based on the local feature because of its efficiency and robustness.

In 2004, Lowe D. G. summarized and formalized the SIFT (Scale Invariable Feature Transform) algorithm [7]. In 2007, Lowe D. G. expanded his previous work, and at the same time presented a panoramic automatic stitching software in the paper [8], which is very robust in image rotation, scaling, and scale transformation. However, because of its large amount of calculations, it is difficult to meet real-time requirements. In 2008, Bay H. proposed the SURF (Speeded Up Robust Features) algorithm [9]. The descriptor of the algorithm has lower complexity than SIFT, which improves the real-time performance of the algorithm. However, in the process of constructing the image of the Pyramid, both use the linear Gauss expansion filters, which causes boundary obscurities and loss of important details [10], thus affecting the accuracy of the feature points. In 2011, Rublee E. proposed the ORB (Oriented FAST and Rotated BRIEF) algorithm [11], which is a fast feature extraction and matching algorithm. It is very quick, but it is less effective in terms of scale. In recent year, many researchers have done a lot of research on image mosaics [12–16]. The A-KAZE algorithm based on nonlinear scale decomposition can solve the above problems. Therefore, we uses A-KAZE algorithm to extract image feature points [17–18], to ensure the real-time and accuracy of feature point extraction and location.

**Fig 1:**
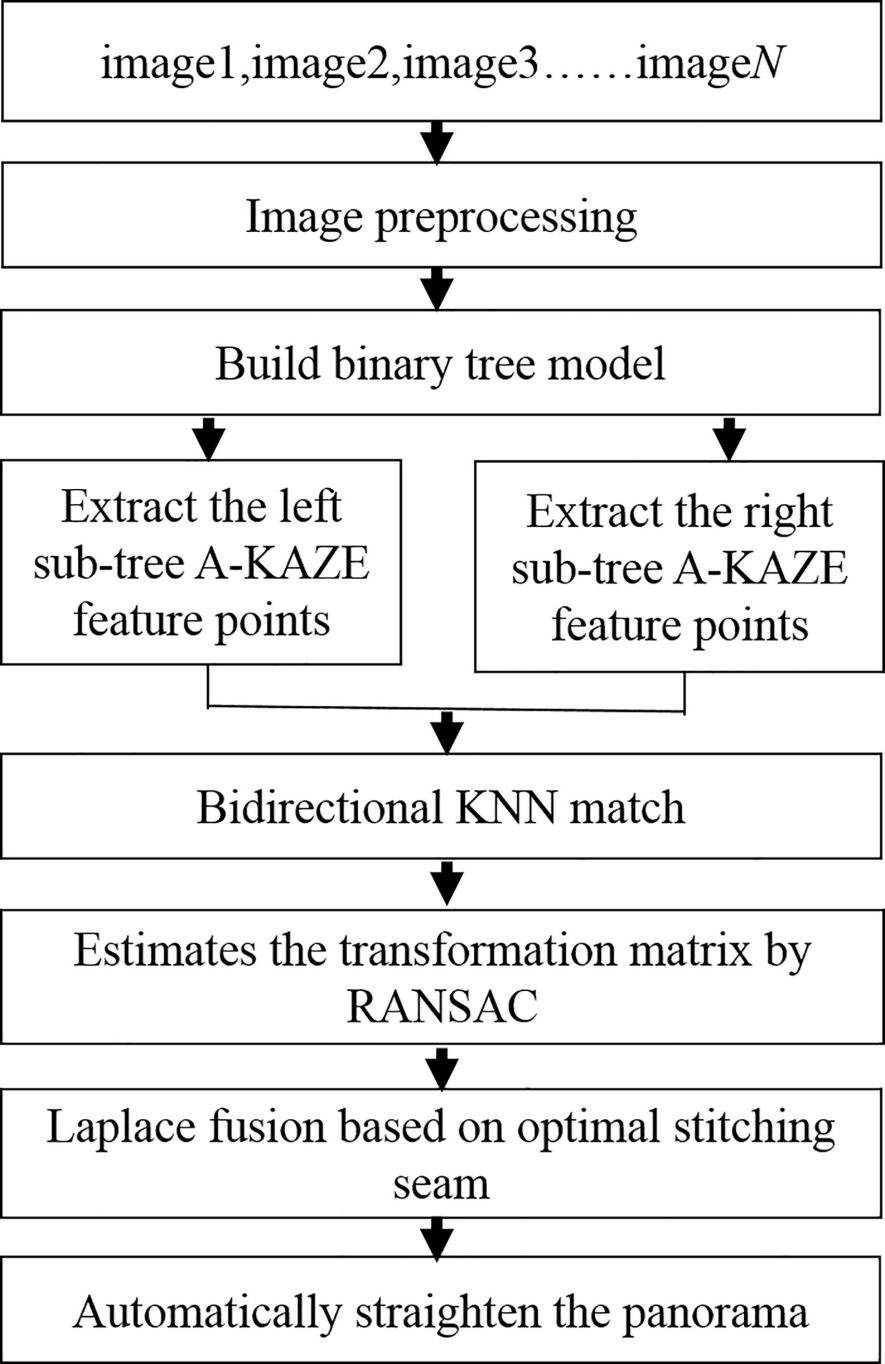
The total process of proposed method.

The image splicing process of Song F. H. takes the first image of the sequence images as the reference image [19], and gradually splices the panorama from left to right. In the case of the large number of input images, the result of the final image splicing is seriously distorted. After that, the authors propose an improved algorithm to select the middle position of the scene as the reference image [20], gradually splicing panorama in the order from the middle to the two sides. Theoretically, the improved method reduces the distortion in half compared with the original method. At the same time, we also use the camera calibration method for image stitching [21]. On this basis, we puts forward the method of image splicing based on the binary tree model. The proposed method takes the input image set as the leaf node set of a binary tree, then uses the bottom-up approach to construct a complete binary tree with the root node image of the binary tree as the ultimate panorama obtained by stitching. We also proposes an automatic image straightening model according to the different degrees of distortion and morphology of the panorama. It has been demonstrated that this method can significantly reduce the distortion of the panorama in the image mosaic of traditional digital image processing. The overall flow chart of proposed method is shown in Fig 1.

The improved image splicing method is shown in the following steps:

Input: *n*(*n*≥2) images sequence *S*(*S*_1_,*S*_2_,…*S*_*n*_) with overlapping regional sequence.

(i) Image preprocessing:

This stage mainly includes image denoising, image geometric correction, color correction and cylindrical projection to facilitate subsequent image stitching.

(ii) Construction of the binary tree model:

According to the properties of the binary tree, there are at most 2^*i*−1^(*i*≥1) nodes in the *i* layer of the non-empty binary tree, the sequence *S*(*S*_1_,*S*_2_,…*S*_*n*_) of the input *n* images is taken as the leaf node set of the binary tree. The number of layers *i* of the binary tree is obtained by the formula $i=\left\lceil \log_{2}^{n}\right\rceil +1$. Construct the binary tree model of *n* images as leaf nodes by the input.

(iii) Image registration of all left and right subtrees in the binary tree model:

(a) The images of the left subtree and the right subtree are respectively *S*_*k*_ and *S*_*k*−1_,*k*∈[1,*n*−1]. Using A-KAZE algorithm to extract the feature points of image *S*_*k*_ and image *S*_*k*−1_, then using bidirectional KNN algorithm to search the matching feature points between *S*_*k*_ and *S*_*k*−1_ according to the shortest Euclidean distance, and the feature point set is stored in array *featureList*.

(b) Calculate the affine transformation model that images *S*_*k*_ to *S*_*k*−1_ based on the matching feature point data set *featureList*, and the result is saved in the array *HList*;

(c) Return steps (a) to continue the image registration of the left subtree and the right subtree until all leaf node image registration is completed.

(iv) According to the array *HList*, use the formula (5) to calculate the affine transformation matrix *H* of the right subtree image to the left subtree image in the leaf nodes of binary tree.

(v) Apply the obtained affine matrix to the right subtree image, so as to the right subtree image has the same coordinate system with the left subtree image.

(vi) Find the optimal splicing between the right subtree image and the left subtree image, and then Laplacian fusion based on optimal stitching seam to achieve the seamless splicing of the two images and get the splicing result graph *ImgResult*.

(vii) Add *ImgResult* to the set *S* to replace the two spliced input images, and then the next splicing process with *ImgResult* is regarded as the new input image.

(viii) Go to step (iv) to perform the leaf node image splicing of the next group of left and right subtrees, until all leaf node images splicing is completed.

(ix) Go to step (ii) to construct the complete binary tree from the next bottom-up recursion, until there is only one image in the set *S*, which is the panoramic view.

(x) Go through the automatic image straightening model to get the panorama corrected.

Output: A panorama that completes the binary tree splicing and improves the distortion error.

## 2. Image registration

### 2.1 A-KAZE feature point extraction

Alcantarilla et al. [17] proposed a new and fast multi-scale feature detection and description algorithm called A-KAZE in 2013. The three main steps to extract the image features in the A-KAZE algorithm include:

(i) Non-linear scale space is constructed by using the principle of nonlinear diffusion filter and the fast explicit diffusion (FED) algorithm to solve implicit difference equations [22–23]. The nonlinear diffusion equation is:

$$\frac{\partial L}{\partial t}=div(c(x,y,t)\cdot \nabla L)$$ (1)

Where *L* is the brightness of the image, *t* is the scale parameter, *div* and ∇ represent the divergence and gradient operators respectively, with *c*(*x*,*y*,*t*) being the conductivity function.

(ii) The feature points of interest are detected. These feature points are in the non-linear scale space and are the local maxima (3×3 pixel field) of the Hessian matrix determinant after scaling. The calculation of Hessian matrix is shown in the following equation:

$$H(L^{i})=\sigma_{i,norm}^{2}(L_{xx}^{i}L_{yy}^{i}-L_{xy}^{i}L_{xy}^{i})$$ (2)

In the formula (2), $\sigma_{i,norm}^{2}$ is the normalized scale factor of the octave of each image in the nonlinear scale. $L_{xx}^{i}$ and $L_{yy}^{i}$, respectively, the horizontal and vertical image of the second-order partial derivative, $L_{xy}^{i}$ is cross-partial derivative.

(iii) The eigenvectors are constructed and the main directions of the eigenvalues are calculated. Based on the first-order differential images, the eigenvectors with scale and rotation invariance are extracted. A-KAZE uses a new kind of binary descriptor M-LDB (Modified-Local Difference Binary) to describe the feature points. We select a patch around the feature point, divide each image patch into *n*×*n* equal-sized grids, and extract representative information from each grid cell. Then, binary test operations on a pair of grid cells (*i* and *j*) are performed. Binary test operation *ϖ* is shown in the following equation:

$$\varpi (Func(i),Func(j))=\{\begin{array}{l} 1,\;if(Func(i)‑Func(j))>0,i\neq j\\ 0,\;otherwise \end{array}$$ (3)

Where *Func*(⋅) represents the function for extracting information from a grid cell.

**Fig 2:**
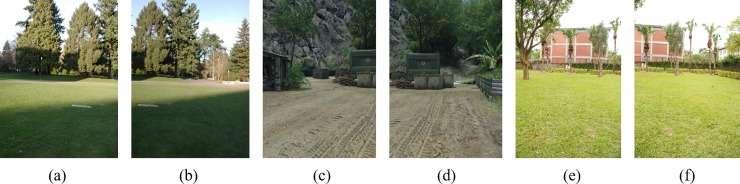
The original image sequence.

**Fig 3:**
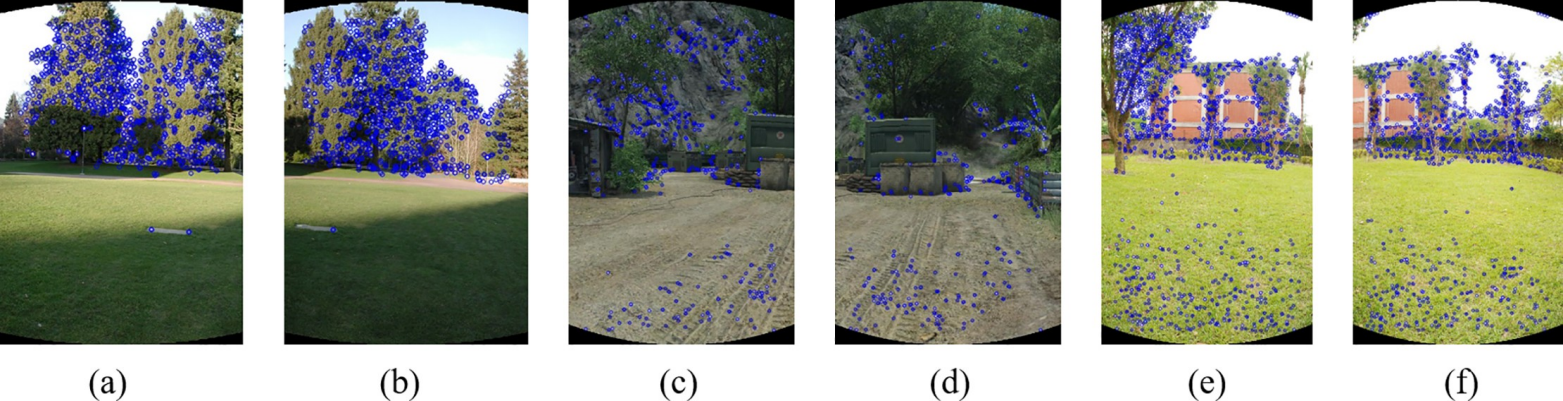
Extract A-KAZE feature points for images in Fig 2.

Fig 3 shows the A-KAZE feature points extracted from Fig 2.

### 2.2 A-KAZE feature registration

After extracting the A-KAZE feature points, two KD-trees are constructed for the reference image and the target image respectively. The next step is taking one of them in turn as the reference for KNN(K Nearest Neighbor) matching, then extract the public matching pairs of matching operations as the initial matching. Finally, the RANSAC algorithm is adopted to remove the outer points and estimate the affine transformation matrix between images.

*r* (*r* = 3) pairs of sets are randomly selected from the *N* matched pairs in the rough matching to estimate the parameters of the affine transformation matrix. The affine matrix structure is shown in the following equation:

$$H=\left[\begin{array}{ccc} h_{11} & h_{12} & h_{13}\\ h_{21} & h_{22} & h_{23}\\ 0 & 0 & 1 \end{array}\right]$$ (4)

where *h*_11_,*h*_12_,*h*_13_,*h*_21_,*h*_22_,*h*_23_ make up the affine transformation matrix.

We choose the matrix *H* as the affine transformation matrix, which corresponds to the maximum number of inliers. The formula for calculating the affine transformation matrix is shown in the following equation:

$$\left[\begin{array}{c} x_{i}^{'}\\ y_{i}^{'}\\ 1 \end{array}\right]=\left[\begin{array}{ccc} h_{11} & h_{12} & h_{13}\\ h_{21} & h_{22} & h_{23}\\ 0 & 0 & 1 \end{array}\right]\left[\begin{array}{c} x\\ y\\ 1 \end{array}\right]$$ (5)

Due to the affine transform matrix having 6 degrees of freedom, 3 pairs of unfair line matching feature points are randomly selected to estimate the transformation matrix.

**Fig 4:**
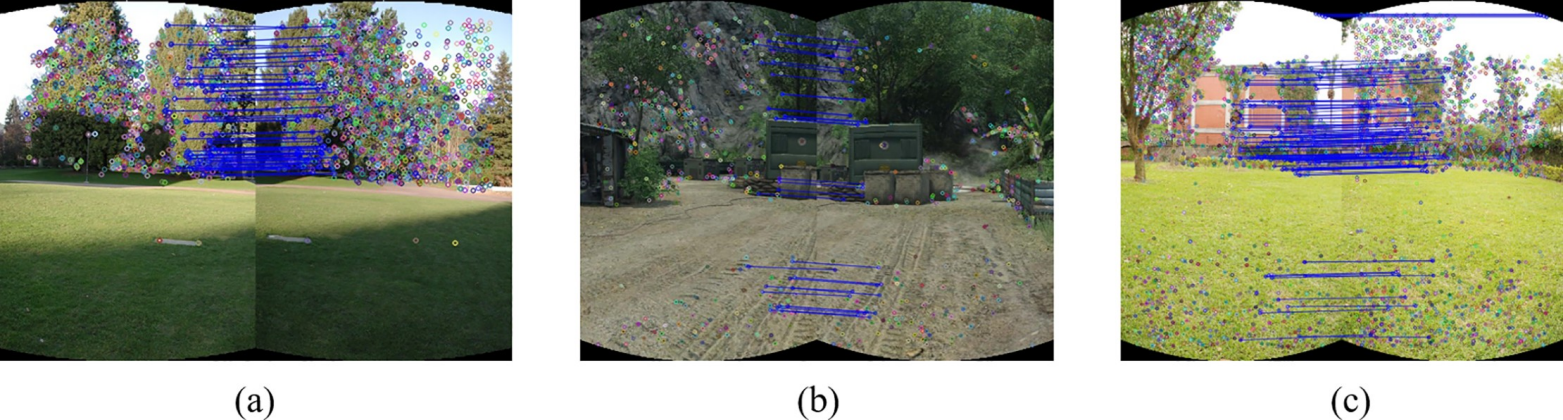
The result of initial matching for images in Fig 3.

**Fig 5:**
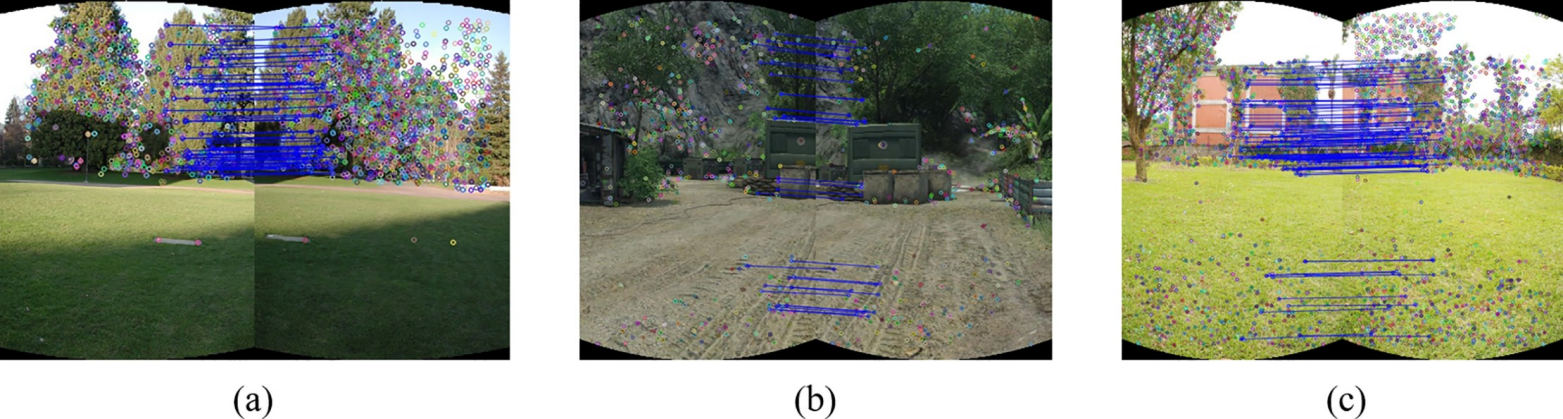
RANSAC for the adjacent images in Fig 4.

Fig 4 shows the initial matching results and Fig 5 shows the matching results after RANSAC excluding false matches.

## 3. Image fusion

### 3.1 Find the optimal stitching line

The goal of image fusion is to automatically transfer the meaningful information contained in multiple source images to a single fused image without information loss [24–26]. After image registration, direct synthesis will lead to discontinuity of color transition [27] and image artifacts appear [28–30] when there are moving objects. So, it is needed to find an optimal stitching line to eliminate the artifacts and hide the image edges [31], which requires the color difference between the two sides of the image to be kept at a minimum and the geometry of the neighborhood to be similar. Therefore, the idea of a dynamic programming method is used to obtain the optimal stitching line with minimum energy. The energy formula is defined as,

$$T(i,j)=\alpha \cdot E_{C}(i,j)+\beta \cdot E_{G}(i,j)$$ (6)

Where *E*_*C*_ represents the color difference in 5×5 rectangular scope around stitching line pixels. *E*_*G*_ represents the change of texture. *α* and *β* are weight values. *α*+*β* is equal to 1. We carried out many experiments of *α* and *β* and found the outcome to be 0.83 and 0.17 respectively.

$$E_{C}=\left\|\frac{1}{5\times 5}\sum_{m=-2}^{2}\sum_{n=-2}^{2}img\_sub(i+m,j+n)-img\_sub(i,j)\right\|$$ (7)

$$\begin{array}{c} E_{G}=sqrt((\frac{1}{5\times 5}\sum_{m=-2}^{2}\sum_{n=-2}^{2}Gradient\_x(i+m,j+n){)}^{2}\\ +(\frac{1}{5\times 5}\sum_{m=-2}^{2}\sum_{n=-2}^{2}Gradient\_y(i+m,j+n){)}^{2})) \end{array}$$ (8)

According to the energy formula, the overlapping points are taken as the starting point *P*, and the three pixels adjacent to the *P* point are taken as the direction of expansion to find the optimal stitching line.

### 3.2 Elimination of the stitching line

In the actual operation, the image mosaic traces still exist. This is due to different shooting angles, which lead to different image exposures.

**Fig 6:**
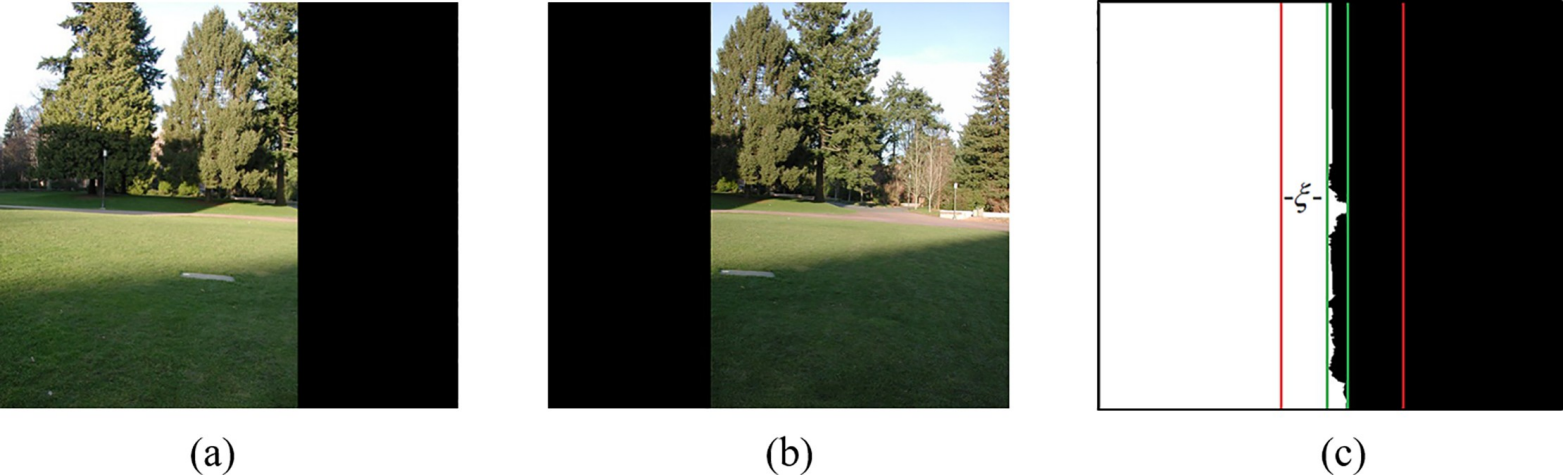
Image before limited by rectangular.

**Fig 7:**
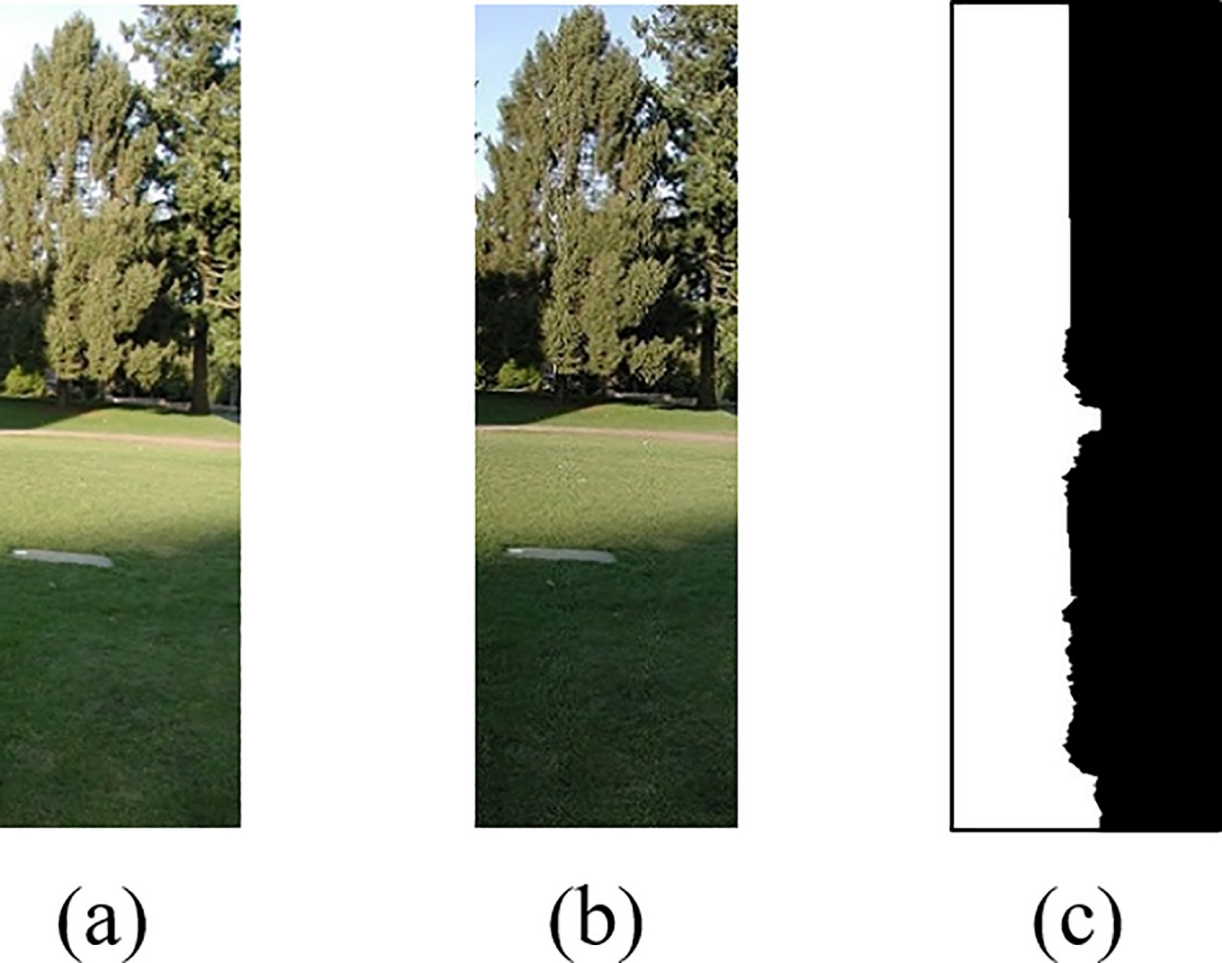
Image limited by rectangular *R*.

For the stitching line to have a natural transition, the method of Laplacian fusion is used to eliminate the stitching line by creating the mask image *I*_*R*_ of the stitching line. The area on the left side of the stitching line is filled with a pixel value of 255, and the right side is filled with a pixel value of 0, as shown in Fig 6(C). The minimum bounding rectangle *R* of the optimal splicing is the area framed by dashed frame, and the left and right boundary of *R* are derived as *x*_min_ and *x*_max_. According to the experimental results, an empirical threshold value *ξ*(*ξ* = 30) is obtained. We take the rectangular area *R*' to make the left boundary as *x*_min_−*ξ*, the right boundary as *x*_max_+*ξ*. Rectangular area *R*' is framed by solid line frame. The steps of Laplacian fusion algorithm are as follows:

(i) The target image *I*_1_ and reference image *I*_2_ after registrations are expanded to the same size as the mask image, and the extended partial pixel values are assigned to 0, as shown in Fig 6(A) and 6(B).

(ii) Through the former step, we obtain three images of the same size as *I*_1_, *I*_2_ and *I*_*R*_, which are shown in (a), (b), (c) in Fig 6, respectively. In these three images, images with limited range of rectangular *R*' are obtained and represented as $I_{1}^{'}$, $I_{2}^{'}$ and $I_{R}^{'}$, which are shown in (a), (b), (c) in Fig 7, respectively.

(iii) Pyramidal decomposition method is used to analyze $I_{1}^{'}$ and $I_{2}^{'}$. *L*_1_ and *L*_2_ are recorded as two images of Laplacian Pyramid. First, the Gauss pyramid of $I_{1}^{'}$ and $I_{2}^{'}$ is built. The construction formula of the Gauss Pyramid is shown in the formula equation:

$$G_{l}(i,j)=\sum_{m=-2}^{2}\sum_{n=-2}^{2}\varpi (m,n)G_{l-1}(2i+m,2j+n)$$ (9)

Where 1≤*l*≤*N*,0≤*i*<*R*_*l*_,0≤*j*<*C*_*l*_, *N* is the top level of the Gauss Pyramid, *R*_*l*_ and *C*_*l*_ are the number of rows and columns of the *i* level of the Gauss Pyramid, respectively. *ϖ*(*m*,*n*) is a two-dimensional separable 5×5 window function. *ϖ*(*m*,*n*) = *h*(*m*)*h(*n*), *h*(⋅) is Gauss density distribution function. The expression of *ϖ*(*m*,*n*) is as the formula equation:

$$\varpi (m,n)=\frac{1}{256}\left[\begin{array}{ccccc} 1 & 4 & 6 & 4 & 1\\ 4 & 16 & 24 & 16 & 4\\ 6 & 24 & 36 & 24 & 6\\ 4 & 16 & 24 & 16 & 4\\ 1 & 4 & 6 & 4 & 1 \end{array}\right]$$ (10)

Then, we build Laplacian Pyramid,

$$\{\begin{array}{c} LP_{l}=G_{l}-G_{l+1}^{*},\;0\leq l<N\\ LP_{N}=G_{N},\;l=N \end{array}$$ (11)

$G_{i+1}^{*}$ is the same size as *G*_*l*_ and is obtained through the Up-sampling from *G*_*l*+1_

(iv) The mask image is processed by Gauss expansion, which makes the area of the stitching line more smooth. Then, we create the Gauss Pyramid of *I*_*R*_ and it is recorded as *G*_*R*_.

(v) According to the specific fusion criteria, the two images $I_{1}^{'}$ and $I_{2}^{'}$ in each layer of Laplacian Pyramid are fused. The formula is as follows,

$$LS_{l}(i,j)=\frac{G_{R_{l}}(i,j)}{255}*L_{1_{l}}(i,j)+\frac{\left(255-G_{R_{l}}(i,j)\right)}{255}*L_{2_{l}}(i,j)$$ (12)

(vi) The fusion image of each layer of Laplacian Pyramid is reconstruct according to the following equation:

$$\{\begin{array}{c} G_{l}=LP_{l}+G_{l+1}^{*},\;0\leq l<N\\ G_{N}=LP_{N},\;l=N \end{array}$$ (13)

**Fig 8:**
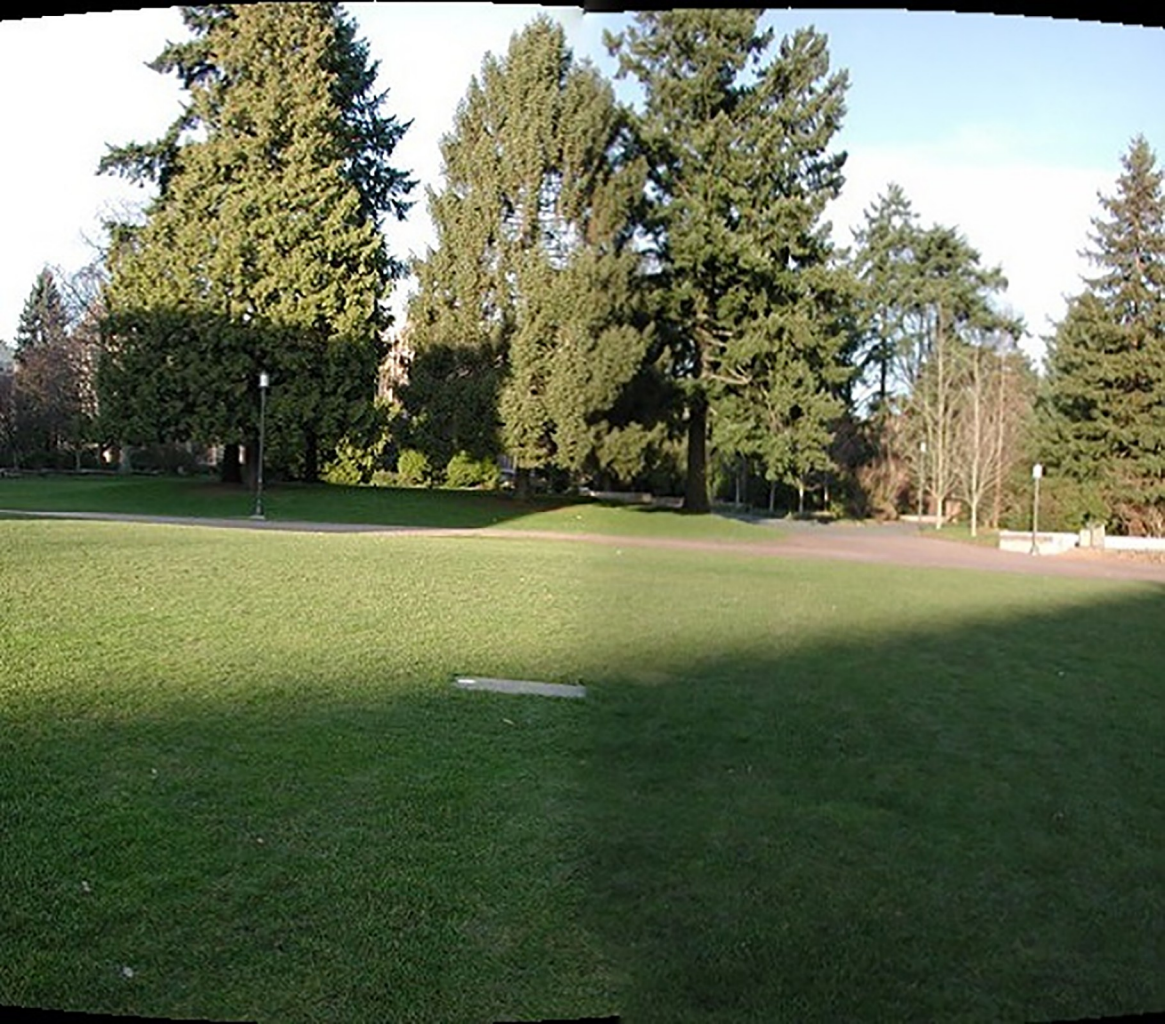
The result after Laplacian fusion.

The experimental results after Laplacian fusion are shown in Fig 8.

## 4. Image mosaic based on binary tree model

Song F. H. [19] use the first image in the sequence as a reference image and each splicing process uses the previous image as a new reference. Therefore, the overlapping area between the new input image and the reference image occupies a smaller proportion of the total reference image area, and the image matching will consume a lot of system resources with very slow splice speed.

**Fig 9:**
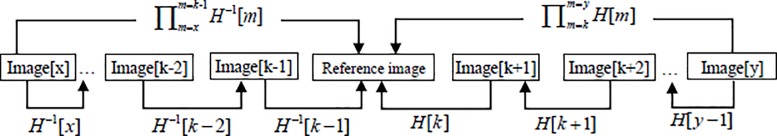
An affine transformation matrix process of mapping an image to the reference image.

After that, reference [20] proposes an improved algorithm to start splicing from the middle of the scene sequence image. The image in the intermediate position is used as the reference image. By calculating the affine transformation matrix *H*[*i*] between adjacent images, the affine transformation matrix of arbitrary position image to a reference image is indirectly obtained. As shown in the formula (14) (*k* stands for intermediate image position index), the next spliced image is dynamically selected according to the number of feature points matching the adjacent image of the statistics. The process of calculating the affine transformation matrix is shown in Fig 9.

$$H=\{\begin{array}{c} \prod_{m=x}^{m=k-1}H^{-1}[m],\;i<k\\ \prod_{m=k}^{m=i}H[m],\;i\geq k \end{array}$$ (14)

**Fig 10:**
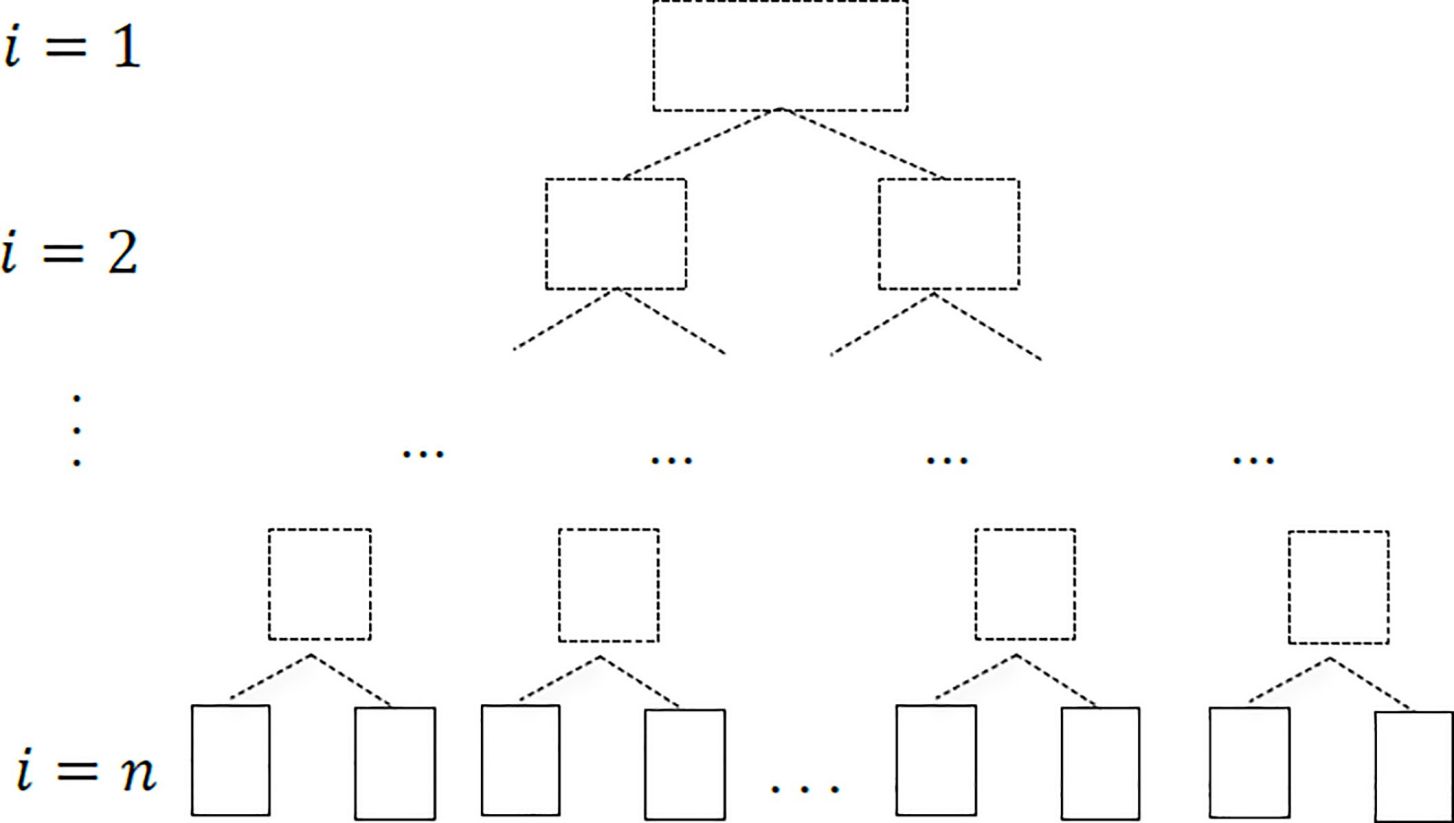
The establishment of binary tree model.

A method of image splicedcing based on binary tree model is proposed. The main idea is to build a new binary tree from the bottom of the leaf, as shown in Fig 10. According to the nature of the binary tree: at the *i*-layer of the non-empty binary tree, there are at most 2^*i*−1^ nodes (*i*≥1), the set of the input *n*(*n*≥2) images is taken as the leaf node set *S*(*S*_1_,*S*_2_,…*S*_*n*_) of the binary tree, and then the number *i* stands for binary tree layers of the *n* image structure is obtained by the following equation:

$$i=\left\lceil \log_{2}^{n}\right\rceil +1$$ (15)

Through the recursive method, the bottom-up method is used to recur *i* times. Each recursion, according to the statistics of the node image matching, the number of feature points to select the splicing of the reference image and construct a complete binary tree, then obtain the binary tree root node, that is, the panorama of multiple images being stitched.

**Fig 11:**
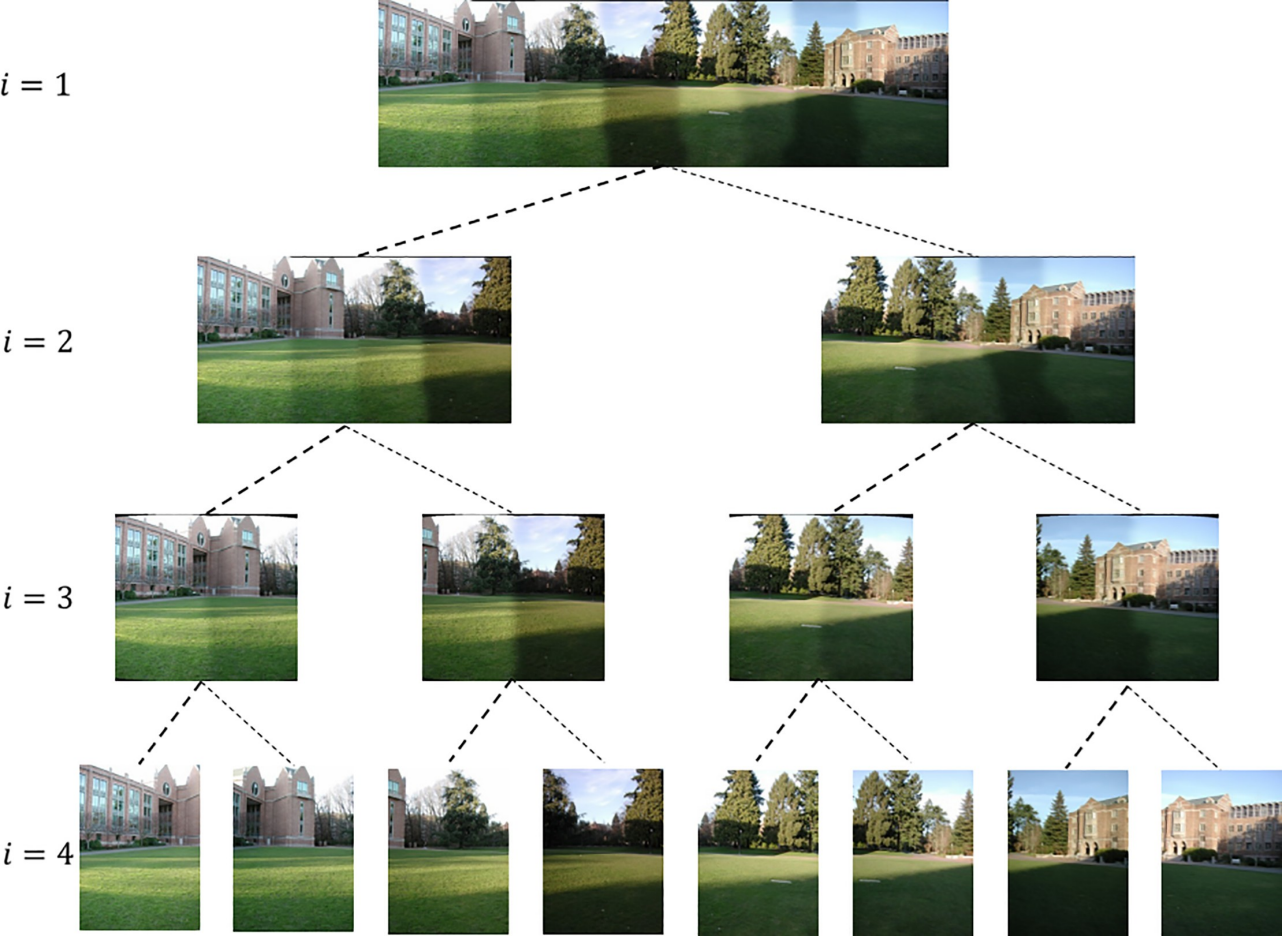
The binary tree image splicing.

In Fig 10, if *n* = 8, that is, the leaf nodes of the $i=\left\lceil \log_{2}^{n}\right\rceil +1$, then *i* = 4 layer represent the input image, and the $i<\left\lceil \log_{2}^{n}\right\rceil +1$ layer is constructed by concatenating the bottom-up recursion. When *i* = 1, the end of the recursion, the root node is the panorama of multiple images being stitched, the experimental results are shown in Fig 11.

## 5. Model of automatic image straightening

**Fig 12:**
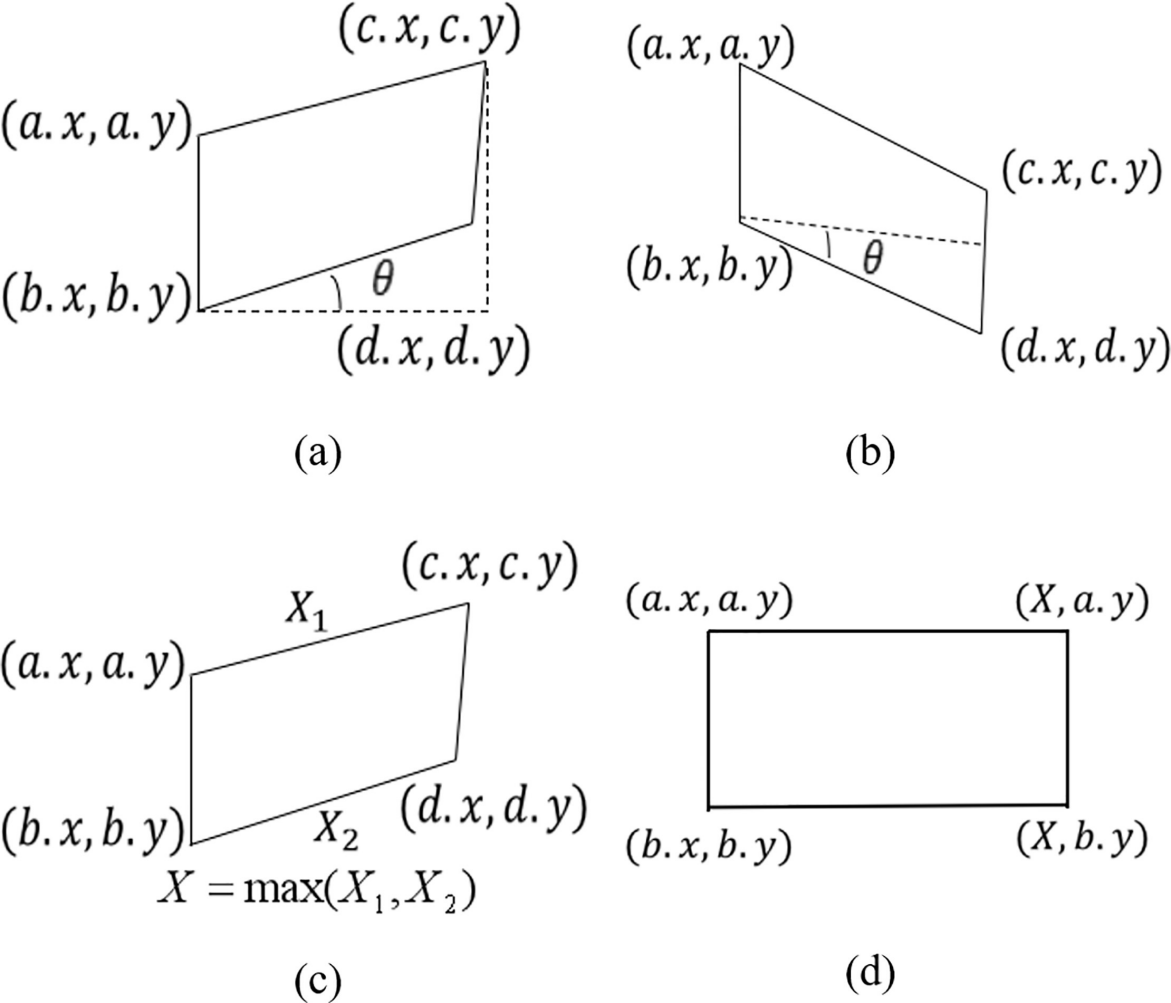
The straightening process.

When splicing multiple image sequences, the oblique distortion occurs due to the accumulation of errors, and it becomes more obvious when the number of input images are increased [32–33]. Therefore, we put forward an automatic image straightening model for the different slanting degree and slanting morphology of the multi-image mosaic. As shown in Fig 12(A) and 12(B), first write down the four vertex coordinates of the top left, bottom left, top right, and bottom right of the panorama, respectively (*a*.*x*,*a*.*y*), (*b*.*x*,*b*.*y*), (*c*.*x*,*c*.*y*) and (*d*.*x*,*d*.*y*). Then the tilt angle of the panorama *θ* is calculated using the triangular anti-tangent formula equation:

$$\theta =\arctan\left(\frac{d.y-b.y}{d.x-b.x}\right)$$ (16)

If $T_{1}<\theta <T_{2},(0\leq T_{1}<T_{2}\leq \frac{\pi }{2})$, *T*_2_,*T*_1_ are the thresholds of the correct value model. After many experiments, *T*_1_ and *T*_2_ are equal to 1° and 10° respectively. Then the value of the panoramic graph is evaluated. As shown in Fig 12(D), set the four vertex coordinates after straightening. The top left and bottom left coordinates are not changed, and the top right and bottom right coordinates are (*X*,*a*.*y*) and (*X*,*b*.*y*). *X* is the length of the pre-estimated panoramic image. Since the panorama before the correction is distorted, the maximum of both *X*_1_ and *X*_2_ is taken as the length of the panorama after the correction.

$$\begin{array}{l} X_{1}=\sqrt{{\left(c.x-a.x\right)}^{2}+{\left(c.y-a.y\right)}^{2}}\\ X_{2}=\sqrt{{\left(d.x-b.x\right)}^{2}+{\left(d.y-b.y\right)}^{2}}\\ X=\max\left(X_{1},X_{2}\right) \end{array}$$ (17)

The perspective transformation matrix can be calculated by four pairs of coordinate points, which can be applied to the whole panorama and the bilinear interpolation can be used to complete the image straightening. The resulting image correction equation is shown in the following equation:

$$S=\{\begin{array}{cc} S, & 0<\theta <T_{1}\\ S\cdot H, & T_{1}\leq \theta \leq T_{2}\\ S, & T_{2}<\theta \end{array}$$ (18)

**Fig 13:**
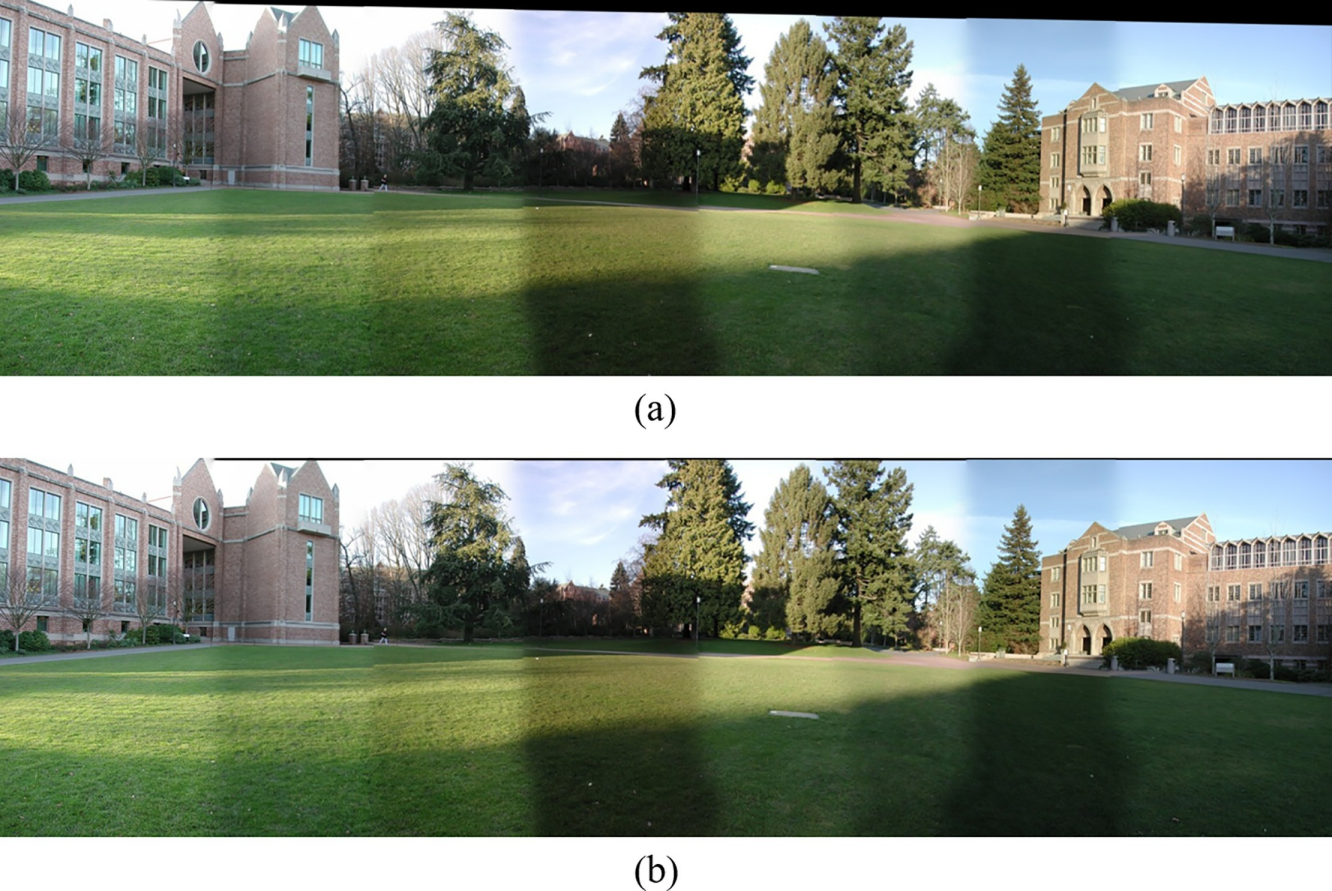
Comparisons of stitching result.

Where *S* is an image matrix and *H* is the perspective transformation matrix. The experimental results are shown in Fig 13.

## 6. The experimental results and analysis

The proposed method is used to experiment with multiple sets of images from multiple scenes. We selected 4 sets of comparative experimental results from multiple experiments to show and analyze in the article. The following are experimental software and hardware environments: CPU: Intel(R) Core(TM) i3-2330M 2.20GHz, OS: Windows 7, Library: OpenCV 3.0.0.

**Fig 14:**
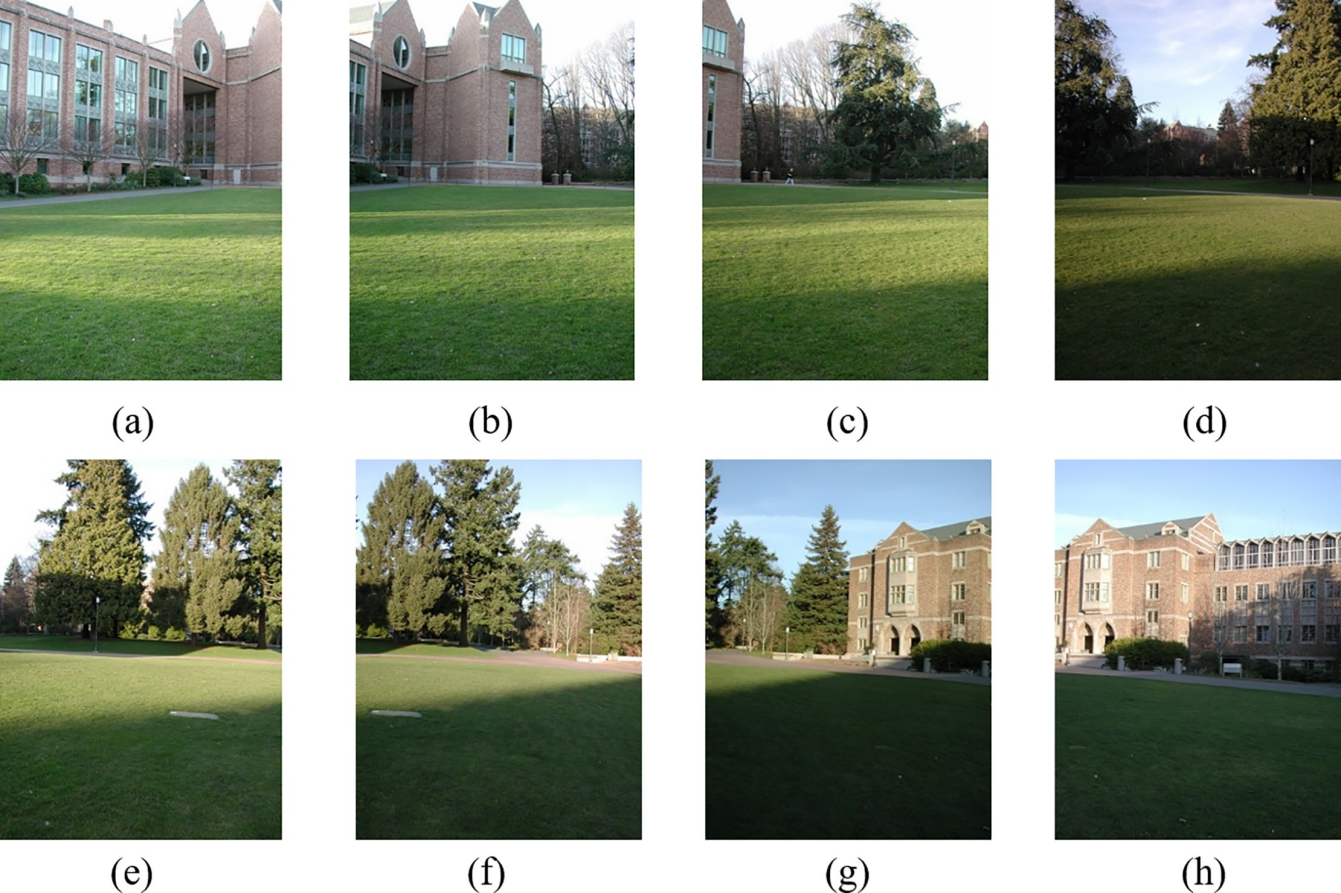
Original image sequences.

**Fig 15:**
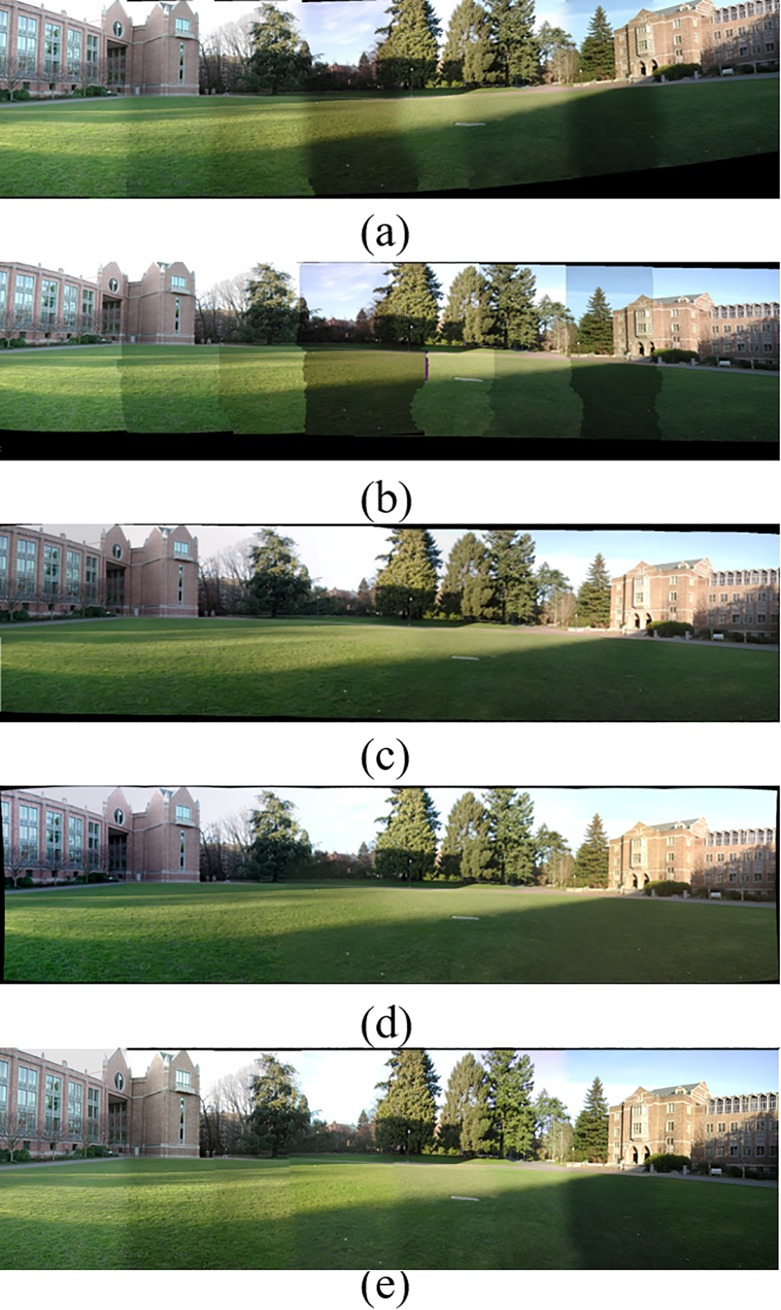
Comparisons of stitching result.

**Fig 16:**
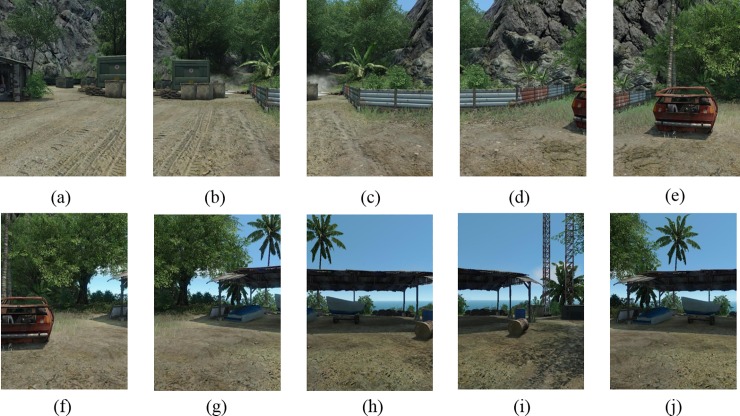
Original image sequences.

**Fig 17:**
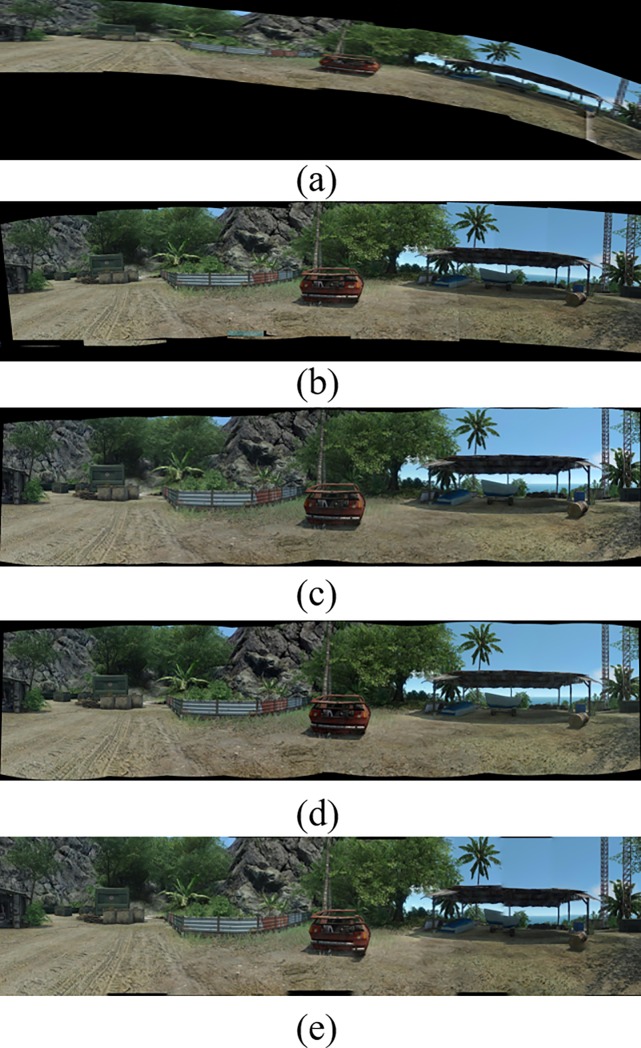
Comparisons of stitching result.

**Fig 18:**
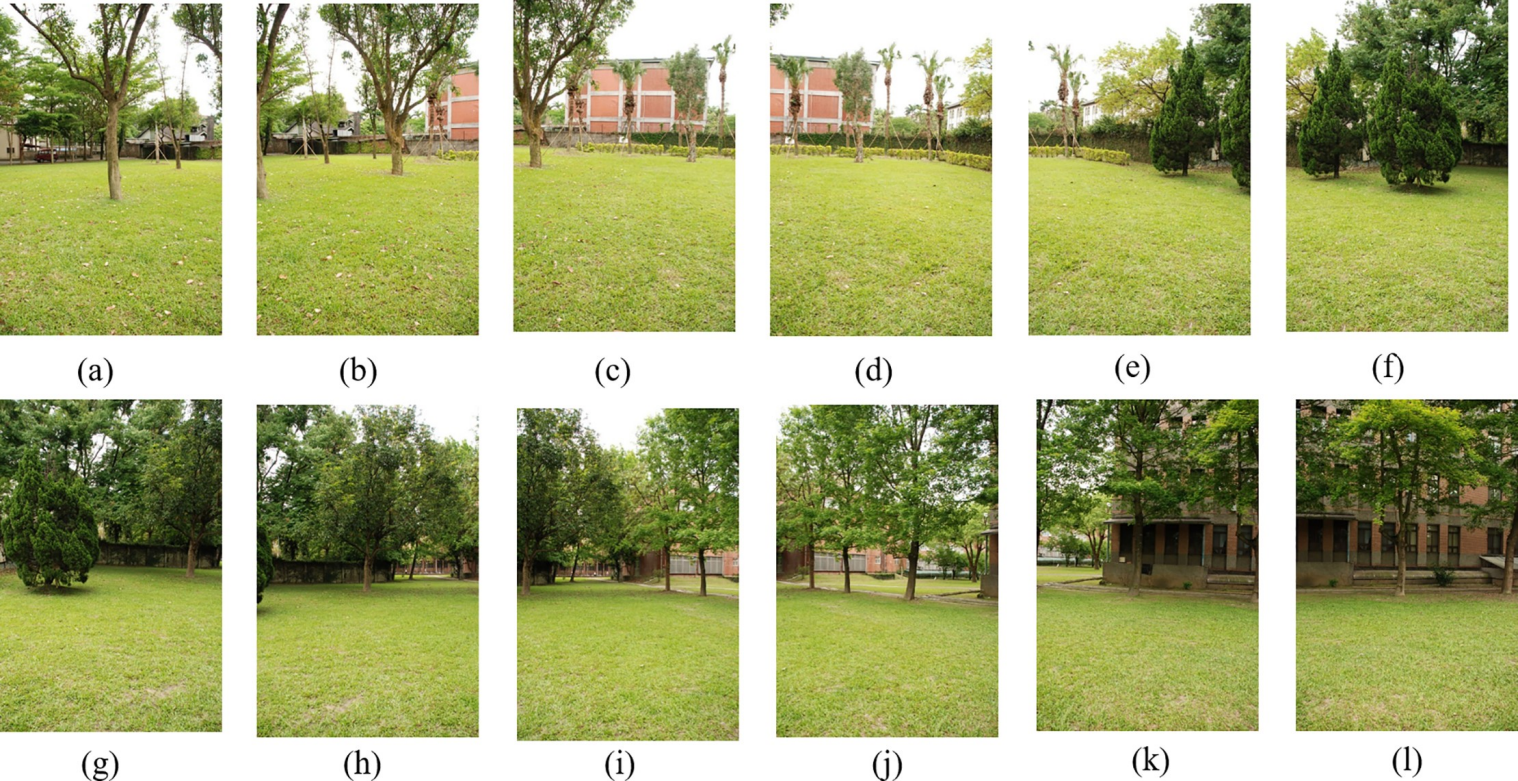
Original image sequences.

**Fig 19:**
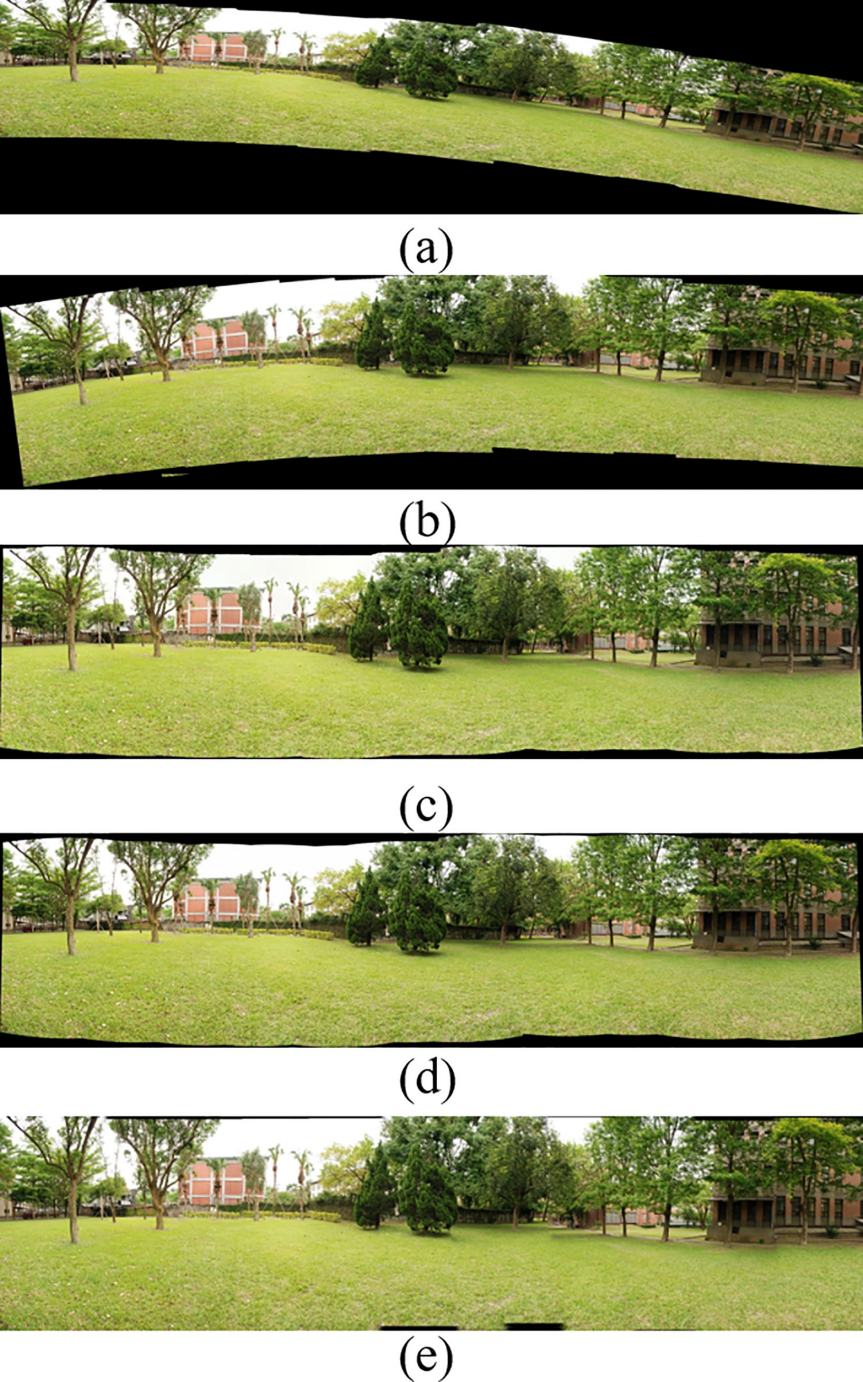
Comparisons of stitching result.

**Fig 20:**
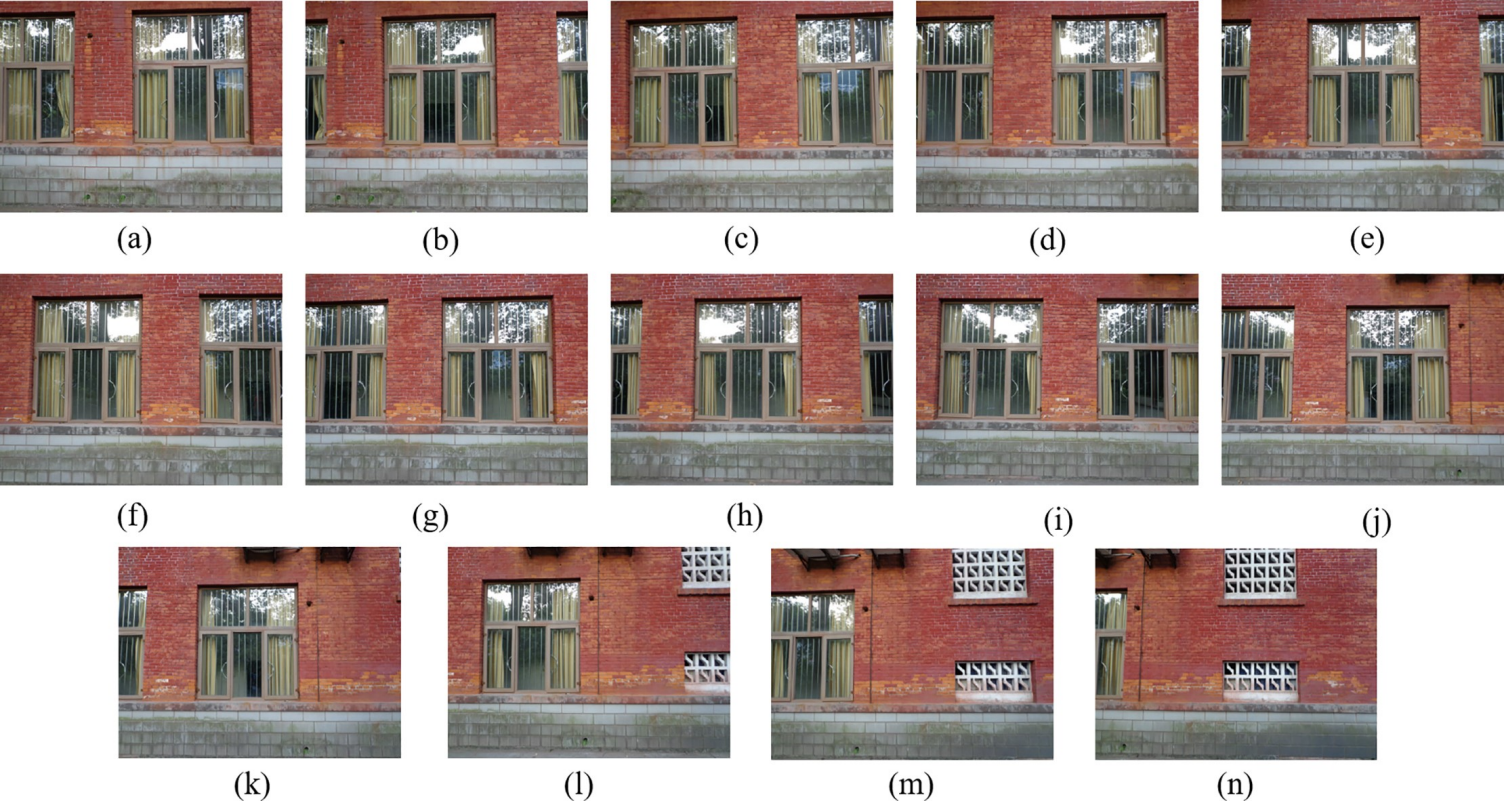
Original image sequences.

**Fig 21:**
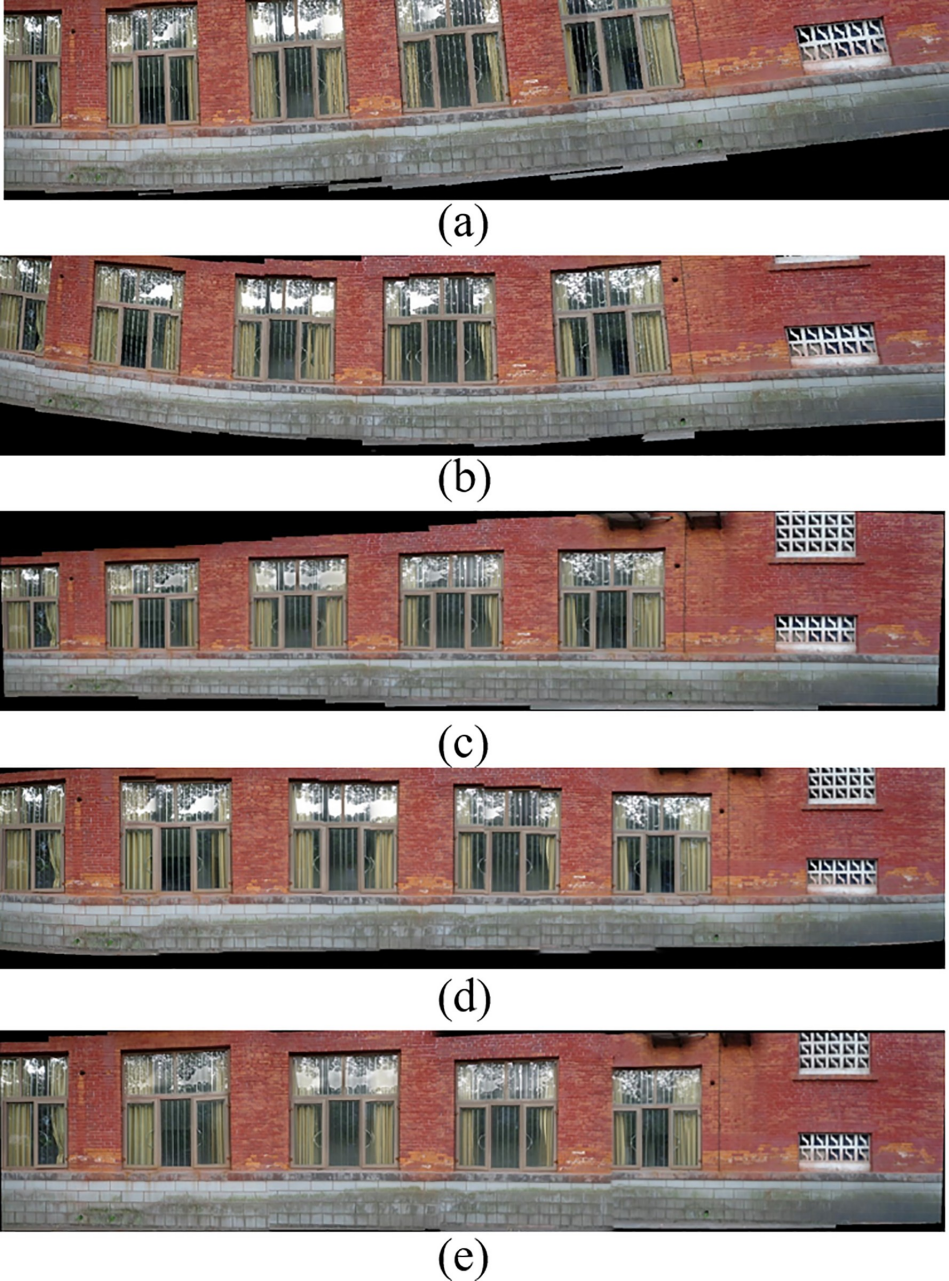
Comparisons of stitching result.

Fig 14 shows 8(384×512) original image sequences, and Fig 15 shows the results of different algorithms splicing the image set of Fig 14. Fig 16 shows 10(500×697) original image sequences, and Fig 17 shows the results of different algorithms splicing the image set of Fig 16. Fig 18 shows 12(500×747) original image sequences, and Fig 19 shows the results of different algorithms splicing the image set of Fig 18. Fig 20 shows 14(800×600) original image sequences, and Fig 21 shows the results of different algorithms splicing the image set of Fig 20. Figs 15(A), 17(A), 19(A) and 21(A) are the results of splicing from left to right [19]. Figs 15(B), 17(B), 19(B) and 21(B) are the results of splicing from the middle to the two sides [20]. Figs 15(C), 17(C), 19(C) and 21(C) are the results based on AutoStitch [8]. Figs 15(D), 17(D), 19(D) and 21(D) are the results based on camera calibration method [21]. Figs 15(E), 17(E), 19(E) and 21(E) are the results of the proposed method splicing. It proves intuitively that the panorama image obtained by the proposed method satisfy the visual requirements for image.

It can be seen from Table 1 that even though the number of feature points extracted by SIFT is larger than that of A-KAZE, the A-KAZE algorithm compares the SIFT algorithm with the same number of feature points. The time cost of A-KAZE significantly is less than the traditional SIFT algorithm.

**Table 1:** Comparison of experimental data for extracting feature points.

Fig	Number of feature points		The time cost of feature extracting (ms)	
	SIFT	A-KAZE	SIFT	A-KAZE
Fig 2(A) 384×512	1440	903	1732	888
Fig 2(B) 384×512	1298	963	1622	922
Fig 2(C) 500×697	4115	610	4160	723
Fig 2(D) 500×697	3902	492	3649	594
Fig 2(E) 500×747	2539	1626	3214	1842
Fig 2(F) 500×747	2156	1249	2777	1435

**Fig 22:**
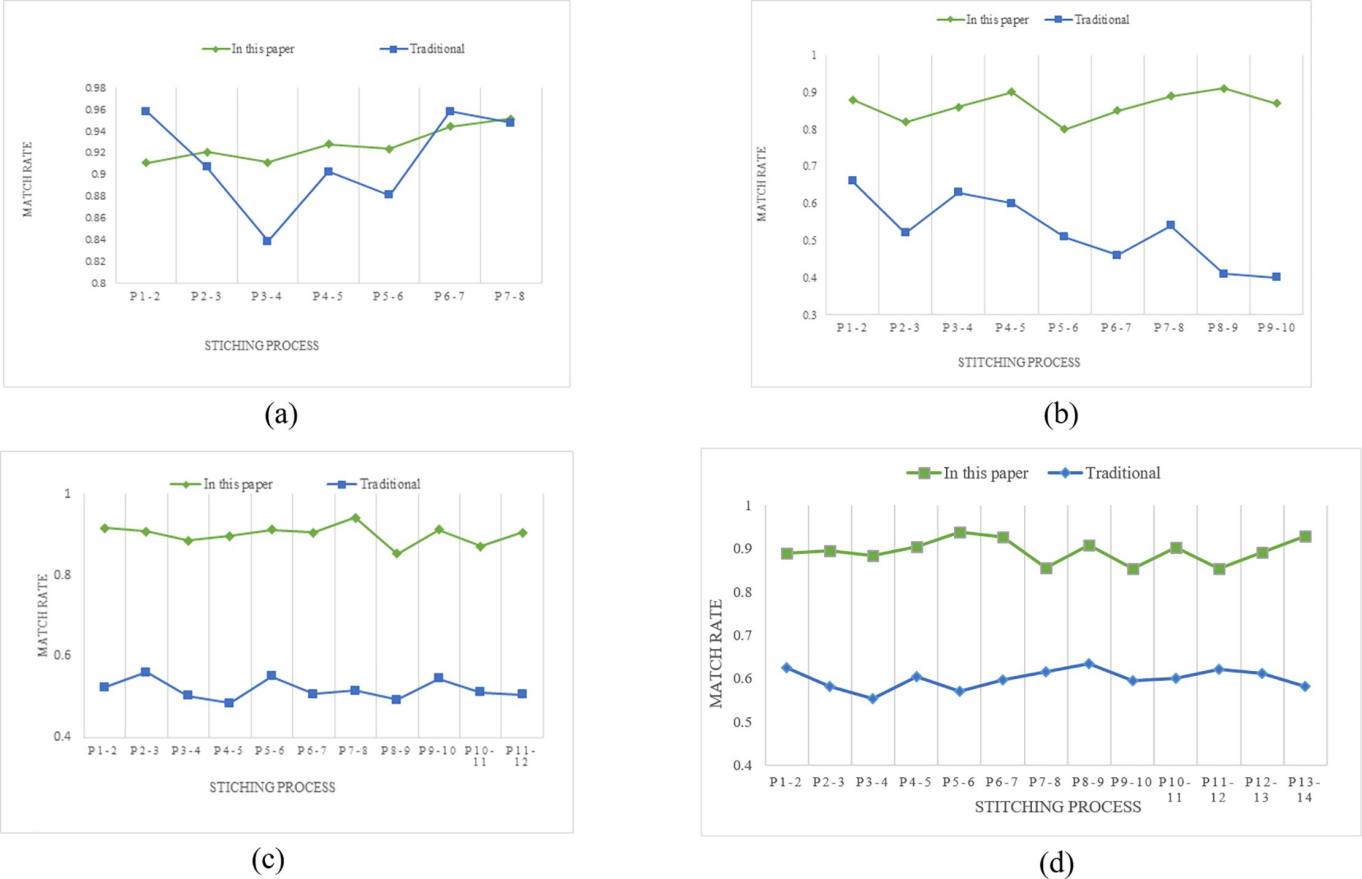
The comparison data of match rate between the traditional SIFT feature matching method and the proposed method.

The correct matching probability of the image is defined in the formula (19). The experimental results are shown in Fig 22.

$$Correct\;match\;rate=\frac{\begin{array}{c} Number\;of\;match\;points\;after\\ eliminating\;mismatching\;points \end{array}}{Number\;of\;first\;match\;points}$$ (19)

Combined with the Fig 22, it can be seen that after RANSAC eliminates the false matching, the proposed method has a higher correct matching rate and a stronger robustness compared to the SIFT feature matching algorithm.

**Fig 23:**
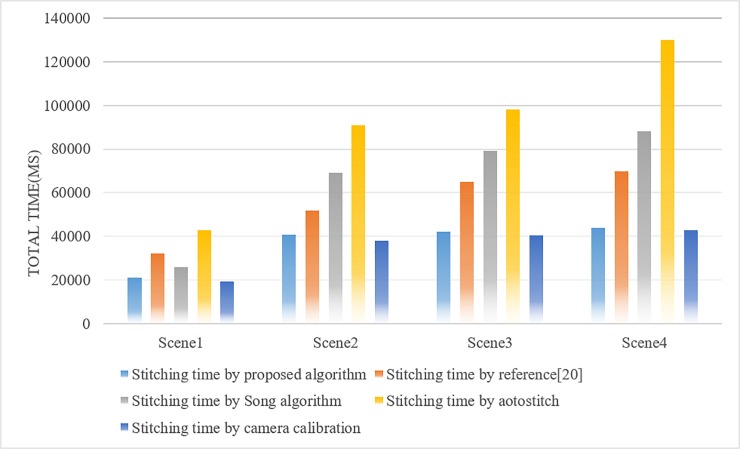
Compare the stitching time of each algorithm.

Fig 23 shows the comparisons of four methods of their mosaic time of the panorama. We can clearly see that the total stitching time of the proposed algorithm is similar to that based on the camera calibration method, and the splicing efficiency of the two algorithms is obviously better than the other three splicing algorithms. But the camera calibration method is based on image block, which make a rough match in the blocked images and a fine match in the most similar blocks by taking advantage of the FAST algorithm. Although the method based on the camera calibration is slightly better in time efficiency than the proposed algorithm, it can be seen from Table 2 and Table 3 that the method based on the camera calibration easily causes a certain degree of distortion in the splicing that resulting in distortion of the panorama. Therefore, for the comprehensive consideration, the proposed algorithm is still superior to the camera calibration method.

**Table 2:** The comparison data of panorama inclination.

Fig	Inclination of panorama from left to right in turn stitching	Inclination of panorama from middle to the two sides stitching	Inclination of panorama stitched by autostitch	Inclination of panorama stitched by camera calibration method	Inclination of panorama obtained by the proposed method
Fig 15	2.46°	1.02°	0.12°	0.05°	0.04°
Fig 17	8.17°	1.11°	0.17°	0.09°	0.07°
Fig 19	5.71°	1.03°	0.15°	0.11°	0.06°
Fig 21	3.44°	1.95°	2.24°	0.92°	0.09°

**Table 3:** The comparison *Info proportion* of panorama.

Fig	*Info proportion* of panorama from left to right in turn stitching	*Info proportion* of panorama from middle to the two sides stitching	*Info proportion* of panorama stitched by autostitch	*Info proportion* of panorama stitched by camera calibration method	*Info proportion* of panorama obtained by the proposed method
Fig 15	92.12%	89.07%	98.17%	97.75%	99.69%
Fig 17	55.82%	82.68%	95.02%	94.16%	99.13%
Fig 19	71.44%	82.59%	96.11%	93.44%	99.27%
Fig 21	85.64%	88.41%	90.72%	91.63%	99.21%

**Fig 24:**
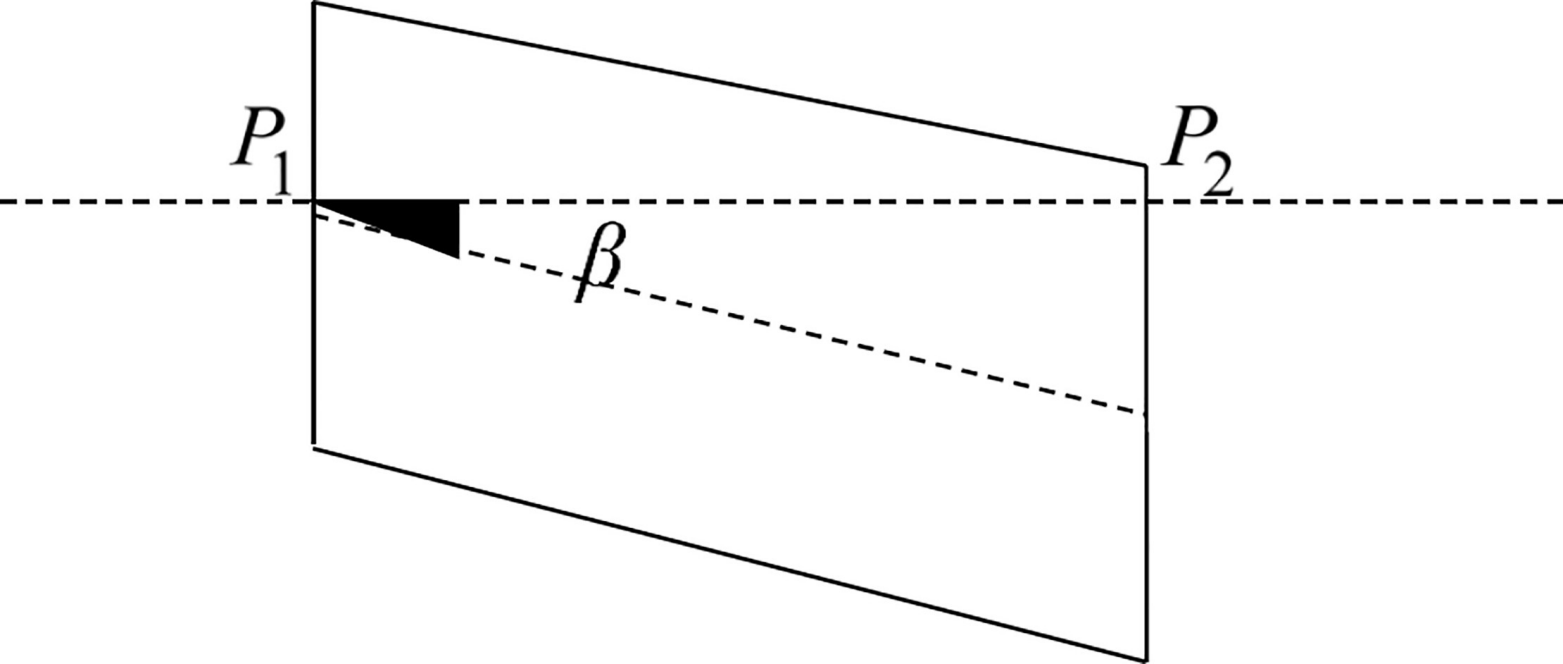
The degree of inclination.

Angle *β* is used to express the distortion degree, as shown in Fig 24. The point *P*_1_ and the point *P*_2_ are the midpoint of the left boundary and the right boundary for the panoramic image respectively. Two points are connected into a straight line and then the angle *β* is obtained between this line and the horizontal line.

It can be seen from Table 1 that the panorama obtained by the algorithm proposed in this paper has almost no distortion. Compared with the other four algorithms, our algorithm is obviously superior to others.

We also define the variable Info proportion, which indicates the ratio of the number of useful pixels in the scene to the total number of pixels of the whole image. The definition is shown in the formula (20), in which width and height represent the size of the panoramic image. The variable Info proportion can also approximately reflect panorama distortion degree.

$$Info\;proportion=1-\frac{black\;pixels}{width\times height}$$ (20)

From Table 2 and Table 3, it can be clearly seen that, as the number of stitched images and the resolution of the images increase, the panoramic image produced by the stitching of the Song algorithm has severe distortion and tilt, which results in a low proportion of information of the panorama. The other three methods, i.e., the splicing algorithm spliced from the middle to the two sides, the method based on the autostitch and the algorithm based on the camera calibration, show the similar results that when the number of stitched images increases, the distortion of the panorama becomes larger, leading to a decrease in the information ratio of the panorama. However, obviously, the algorithm proposed in this paper is almost unaffected by the number of images and resolution. Therefore, our algorithm can obtain high-quality panoramic images, which greatly improves the panoramic distortion phenomenon.

## 7. Conclusions

We presents an image stitching method based on the binary tree, which solves the problems of obscure boundary and detail loss. In addition, the improved method accelerates the panoramic stitching time efficiency and obtains a high-resolution, high-quality panorama image. Being different from the traditional stitching method, the improved method changes the process of selecting the reference image and puts forward a method of image selection based on the binary tree model, which takes the input image set as the leaf node set of binary tree. Then by using the bottom-up approach to construct a complete binary tree, the root node image of the binary tree is the ultimate panorama obtained by stitching. Meanwhile, the improved method proposes an automatic image straightening model to rectify the panorama, which further improves the panoramic distortion. The experimental results show that the proposed method improves the efficiency of splicing, enhances the robustness of feature points matching, and greatly improves the panoramic distortions.

## Supporting information

S1 Fig The experimental images provided by the author are collected in minimal underlying data set.rar. (RAR)
